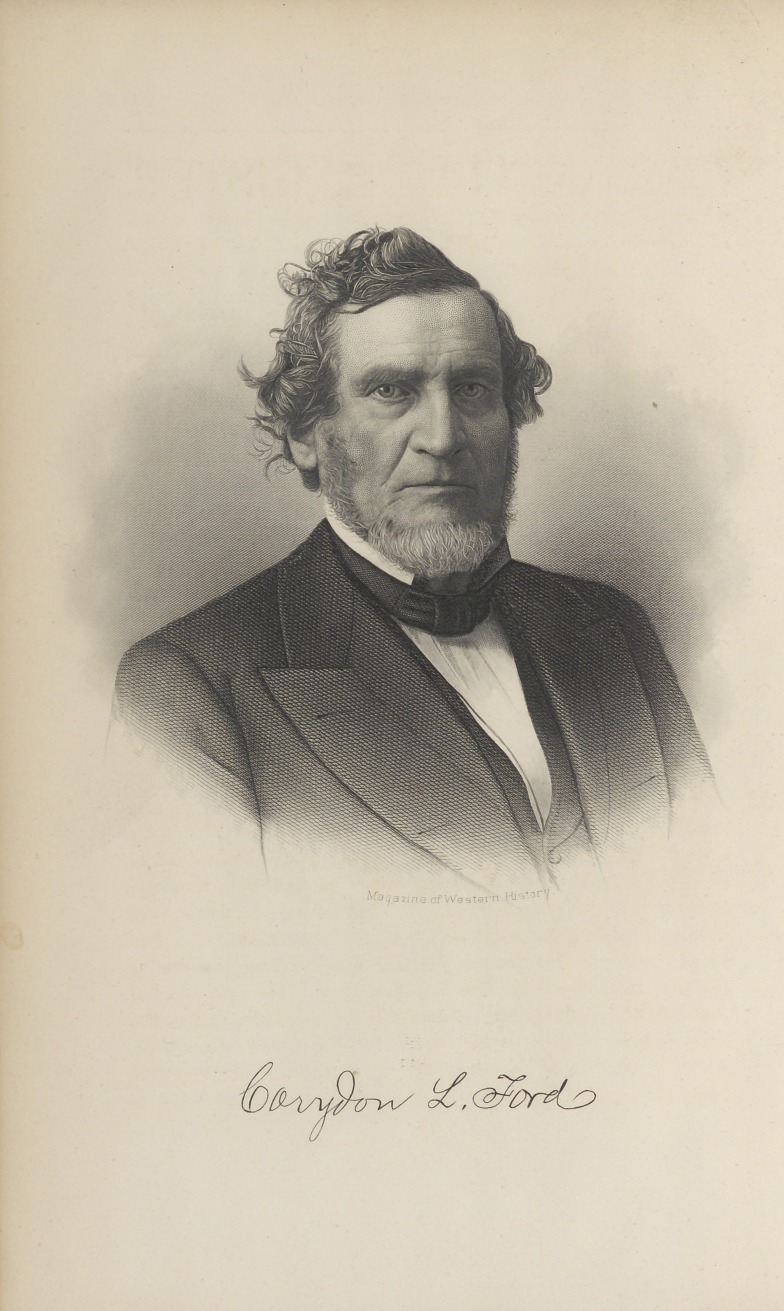# Corydon L. Ford, L.L.D., M.D., D.D.S.

**Published:** 1887-01

**Authors:** 


### THE DENTAL REGISTER.

VOL. XLI.] JANUARY, 1887. [No. 1.

### COMMUNICATIONS.

### Corydon L. Ford, L.L.D., M.D., D.D.S., of the University of Michigan.

Corydon L. Ford, A.M., L.L.D., M.D., D.D.S., was born in Lexington, Green County, New York, August 29th, 1813. His parents were Abner and Catherine Frint Ford, who were natives of New York, and of English descent. They removed to Otsego County, New York, when the subject of this sketch was one year old, where he resided until he was twenty-one years of age. He was a delicate boy, and unable to follow successfully the occupation of a farmer, which was the business of his father; and, after obtaining a common school education, engaged, at the age of seventeen, in teaching a district school in his neighborhood, at nine dollars per month and board.

When he attained his majority, he removed to the western part of his State, and commenced the study of medicine. After a time, feeling the need of a more thorough elementary education, he entered Canandaigua Academy, where he completed an academical course, and then returned to his medical studies, and became a student of Dr. Edson Carr, of Canandaigua, who combined the practice of dentistry with general medical practice, as was not unfrequently tbe case fifty years ago. Here he acquired such knowledge of dentistry as enabled him to combine dental with such general practice as his health enabled him to engage in, while not on duty as Demonstrator of Anatomy, until the spring of 1849. In 1842, January 25th, he graduated in medicine at

Geneva Medical College, where he had developed such a taste and aptitude for anatomical pursuits, under the instruction of Professor James Webster, that, on the day of his graduation, he was appointed Demonstrator of Anatomy in that school. He served in that capacity with extraordinary success till January, 1849. In 1846 Buffalo Medical College was organized, when he was called to the same duties in that institution. There he served with Flint, Hamilton, White, and others, until 1849, when he was appointed Professor of Anatomy in Castleton Medical College, Vermont, where he continued to lecture until the college was suspended, in 1862. The honorary degree of A.M. was conferred upon him by Middlebury College, in 1856. In 1854 he was appointed Professor of Anatomy in the University of Michigan, where he has continued his annual course of lecturers and instruction on Anatomy, and much of the time on Physiology also, for the last thirty-two years. In 1860 he was appointed Professor of Anatomy in Berkshire Medical College, Pittsfield, Massachusetts, where he lectured for several years, at a season of the year, when the University of Michigan was not in session. In 1864 he accepted the Chair of Anatomy in the Medical School of Maine, connected with Bowdoin College, at Brunswick. This position he held until 1870, when he took a recess from his labors in Europe. His last appointment was to the Chair of Anatomy in Long Island College Hospital Medical School, Brooklyn, New York, which position he has held for the last nineteen years, giving his course there in the intervals of his work in the University of Michigan, at Ann Arbor, where he resides. Neither Professor Ford's tastes nor physical condition have inclined him to engage in the general practice of medicine or surgery, though he has, from time to time, performed operations, with the skill and success which might be expected from so expert an Anatomist; but almost his entire time and energy, for the long period of forty-four years, have been devoted to teaching his favorite science. No man, in this country at least, has taught Anatomy to a greater number of students; and no one in any country, has taught better, more faithfully, and inspired more enthusiasm.

From his genial manner, as well as from his superior skill, the labor of no one in the same field has been more fully appreciated than that of Professor Ford. Professor Ford has prepared a manual of questions on Anatomy, Histology, and Physiology, which his many pupils highly prize as an important aid in the study of those subjects. In 1878 he was made a member of the Dental College Faculty, and has given special instruction to dental students, dwelling at greater length, and with more minutiæ, on topics that more directly concern the dental practitioner. In aid of this work he has prepared questions on Anatomy and Physiology of the teeth and associated part for dental students. With the view of awakening an interest in a subject closely allied to the work of the dentist, he has prepared a syllabus of lectures and questions on Odontology, human and comparative, for students of the Dental College. Up to the present time his work in the University has been confined to the six months following October 1st, after which he has lectured at the College Hospital, in Brooklyn, New York. An industrious life and advancing years sufficiently indicate the propriety of lessening the amount of work in his department, and he has recently resigned his position at Long Island College, and will henceforth confine his labors to the University of Michigan. In 1881 he was honored by the University with the title of L.L.D. During the brief existance of the New York College of Dentistry, at Syracuse, Dr. Ford was connected with it and gave instructions during the years 1852-3 and 1853-4, where he received the title of D.D.S., conferred by the institution where he was thus engaged, and from which he resigned upon his appointment to the University of Michigan in June, 1854. Thus it will be seen that Dr. Ford has been engaged in teaching Anatomy, his favorite study, for over forty years. Investigation would perhaps discover that his name is on as many diplomas as any American teacher. He has given one hundred and one courses of lectures on Anatomy besides all his other work.

Magazine of Western History.

Corydon L. Ford

## Figures and Tables

**Figure f1:**